# Transcriptomic Analysis of *Petunia hybrida* in Response to Salt Stress Using High Throughput RNA Sequencing

**DOI:** 10.1371/journal.pone.0094651

**Published:** 2014-04-10

**Authors:** Gonzalo H. Villarino, Aureliano Bombarely, James J. Giovannoni, Michael J. Scanlon, Neil S. Mattson

**Affiliations:** 1 Department of Horticulture, Cornell University, Ithaca, New York, United States of America; 2 Department of Plant Biology, Cornell University, Ithaca, New York, United States of America; 3 Boyce Thompson Institute for Plant Research, Cornell University, Ithaca, New York, United States of America; 4 Robert W. Holley Research Center for Agriculture and Health, USDA-ARS, Ithaca, New York, United States of America; University of Innsbruck, Austria

## Abstract

Salinity and drought stress are the primary cause of crop losses worldwide. In sodic saline soils sodium chloride (NaCl) disrupts normal plant growth and development. The complex interactions of plant systems with abiotic stress have made RNA sequencing a more holistic and appealing approach to study transcriptome level responses in a single cell and/or tissue. In this work, we determined the *Petunia* transcriptome response to NaCl stress by sequencing leaf samples and assembling 196 million Illumina reads with Trinity software. Using our reference transcriptome we identified more than 7,000 genes that were differentially expressed within 24 h of acute NaCl stress. The proposed transcriptome can also be used as an excellent tool for biological and bioinformatics in the absence of an available *Petunia* genome and it is available at the SOL Genomics Network (SGN) http://solgenomics.net. Genes related to regulation of reactive oxygen species, transport, and signal transductions as well as novel and undescribed transcripts were among those differentially expressed in response to salt stress. The candidate genes identified in this study can be applied as markers for breeding or to genetically engineer plants to enhance salt tolerance. Gene Ontology analyses indicated that most of the NaCl damage happened at 24 h inducing genotoxicity, affecting transport and organelles due to the high concentration of Na^+^ ions. Finally, we report a modification to the library preparation protocol whereby cDNA samples were bar-coded with non-HPLC purified primers, without affecting the quality and quantity of the RNA-seq data. The methodological improvement presented here could substantially reduce the cost of sample preparation for future high-throughput RNA sequencing experiments.

## Introduction

Abiotic stress is the negative effect on living organisms of non-living factors such as high temperature, drought and salinity. Abiotic stress affects normal plant growth and development and severely reduces agricultural productivity. Abiotic stressors, especially salinity and drought, are the primary cause of crop loss worldwide, leading to 50% average yield reductions per year for major crops [1], [2].

Due to the important role of the Solanaceae family in agronomic and ornamental crops, holistic-scale approaches have been used to examine salt tolerance in this family. Root proteomic profiling in four tomato (*Solanum lycopersicum*) accessions (Roma, Super Marmande, Cervil and Levovil) was conducted in response to short-term stress by exposing hydroponically grown plants to 100 mM NaCl [3], and a cDNA microarray was used on two cultivated tomato genotypes (LA2711 and ZS-5) growing hydroponically under 150 mM NaCl to study gene expression in early stages of development in tomato plants [4].

RNA-seq offers several advantages over existing technologies; it requires neither previous genome annotation nor pre-synthesized nucleotide as probes and it is not limited by Expressed Sequence Tag (EST) availability [5]. Transcriptome sequences can be reconstructed by *de novo* assembling millions of short DNA sequences (reads) [6] enabling downstream analysis such as novel gene discovery or expression profile analysis [7], [8]. The assembly of DNA reads into a meaningful transcriptome can be performed with different *de novo* assemblers such as Trinity [9], Trans-ABySS [10], and SOAPdenovo-trans [11]. Thus, RNA-seq has become the method of choice to carry out transcriptomic analysis in both model and non-model organisms [12].

*De novo* transcriptomes have been successfully performed through the Illumina platform in a variety of non-model species, including *Lupinus albus* (lupin) [13], *Cicer arietinum* (chickpea) [14], *Ipomoea batatas* (sweetpotato) [15] and *Medicago sativa* (alfalfa) [16], to name a few. Zenoni *et al.* (2011) used 454 sequencing to generate *de novo* assembled transcriptomes separately for *Petunia axillaris* and *Petunia inflata*, parental species of *Petunia hybrida,* to develop microarray chips for transcriptomic analyses to study seed coat defects in a *P. hybrida* mutant [17]. Paired-end read sequencing libraries are widely used in transcriptomic studies to reduce the occurrence of *de novo* mis-assembled reads into artificial contig sequences and chimeras [18], and strand-specific libraries improves RNA-seq by accurately identifying antisense transcripts and boundaries of closely situated genes [19].

The objective of this study was to carry out the first, to our knowledge, whole-transcriptome expression profiles of transcripts through RNA-seq in any Solanaceae plant grown under salinity conditions. Utilizing our newly developed gene index and expression patterns, we identified new candidate genes whose expressions are highly induced as a response to NaCl. We hypothesized that plant response will parallel drought stress in the short term (6 h) and in the longer term (24 h) plant response will be directed to control ion uptake and eliminating toxic ion concentration in the cytoplasm. We hypothesize that short term responses should evidence the up-regulation of Heat Shock Proteins, stress hormones (ABA, ethylene) and signaling transduction components. In this work we also present the most in-depth *Petunia hybrida* reference transcriptome by paired-end sequencing cDNA libraries. The novel transcriptome, available at the SOL Genomics Network (SGN) http://solgenomics.net [20], can be used as an excellent tool for biological and bioinformatic inferences in the absence of an available *Petunia* genome.

Transcriptomic gene expression has shed light on novel salt stress mechanisms and differentially expressed genes related to salt stress previously undescribed. While the predominant focus of our work is on transcriptomic analyses for salt stress, a secondary objective was to test the utility of a cost saving modification for RNA-seq library construction with non-HPLC purified primers, which has the potential to greatly reduce the cost of library preparation for future RNA-seq-based-experiments.

## Materials and Methods

### Plant material and salt treatments

*Petunia x hybrida* cv. ‘Mitchell Diploid’ were germinated in a soilless substrate (Metromix 280, Sun Gro Horticulture LTD.,Vancouver, Canada) for 3 weeks. After seedlings were ca. 8 cm tall and well rooted, 60 seedlings were selected for uniformity. Roots were washed to remove substrate and seedlings were secured in rockwool around the stem base and placed into 4 L containers in solution culture (one plant per container). The nutrient solution used was a modified Hoagland's solution (4 mM KNO_3_, 1 mM MgSO_4_, 1 mM NH_4_H2PO_4_, 4 mM Ca(NO_3_)_2_•4H_2_O, 18 μM Fe-EDDHA, 2 μM CuSO_4_•5H_2_O, 4 μM ZnSO_4_•7H_2_O, 0.2 μM H_2_MoO_4_•H_2_O, 28 μM MnCl_2_•4H_2_O, 4 μM H_3_BO_3_) prepared in reverse osmosis filtered water. The solution was kept aerated by continuously bubbling air into each container using an aquarium pump to maintain oxygen saturation. After 1 week of establishment in the hydroponic systems, 20 containers were selected for uniformity and transferred to a growth chamber (200 μmol light 12 h/d, 22°C day/night and 45% relative humidity). The 20 plants were selected based on phenotype (similar size, number of branches, height, and absence of nutritional or biotic disorders), and developmental stage (first flower initiation). After one week of growth chamber acclimation, the two least representative plants for each treatment were discarded from the experiment. The remaining eighteen plants were randomly divided into two groups of nine containers. The control group received the Hoagland's solution with no added NaCl, the salt treatment group received Hoagland's solution amended with 150 mM NaCl. Containers were distributed randomly throughout the growth chamber.

### Tissue sample and RNA isolation

To reduce plant-to-plant variability, we established three groups of three randomly selected plants within each treatment condition. Tissue samples from the three plants per group were pooled together to create one biological replicate. At each time point, the most recently expanded leaf (the fourth or fifth leaf from the lateral meristem) from a lateral branch was selected. Plant leaves were sampled at 0, 6, and 24 h after salt treatment was applied. Therefore, for each time point six biological replicates were collected (3 from control and 3 from salt treatment) resulting in 18 samples total. To reduce the number of samples for RNA-seq, only the control samples were used at time point 0 (just prior to initiation of salt stress) which yielded 15 samples for the experiment. Samples were immediately frozen in liquid nitrogen and stored at −80°C prior to RNA isolation. Total RNA was isolated using Trizol Reagent (Invitrogen, USA) and purified through a Qiagen RNeasy Column (Qiagen, Germany) according to the manufacturer's instructions. A 1% agarose gel buffered by Tris–acetate–EDTA was run to indicate the integrity of the RNA. Seven samples were further quantified in an Agilent 2100 Bioanalyzer (Agilent, Santa Clara, CA, USA) at the Core Laboratories Center Genomics, Institute of Biotechnology, Cornell University (http://www.biotech.cornell.edu/biotechnology-resource-center-brc) to verify total RNA quality. RNA Integrity Number (RIN) for the samples analyzed were 8.5, 9.1, 8.9, 8.5, 8.5, 8.7 and 6.7.

### Library preparation and sequencing

Libraries corresponding to three biological replicates from each time point plus treatment combination (control time 0 h, control and NaCl time 6 h and 24 h) were constructed following a High-Throughput Illumina Strand-Specific RNA Sequencing Library protocol [21]. Briefly, 2–5 μg of total RNA was used for polyA RNA capture with magnetic oligo(dT) beads (Invitrogen, USA), fragmented at 95°C for 5 min and eluted from beads. Cleaved RNA fragments were primed with random hexamer primers to synthesize the first cDNA strand using reverse transcriptase SuperScript III (Invitrogen, USA) with dNTP. The second cDNA strand was generated by DNA polymerase I (Enzymatics, USA) with dUTP mix. Following end-repair (Enzymatics, USA), dA-tailing (Klenow 3′–5′, Enzymatics, USA) and adapter ligation (T4 DNA Ligase HC Enzymatics, USA), the second dUTP-strand was digested by uracil DNA glycosylase (Uracil DNA Glycosylase, Enzymatics, USA). The resulting paired-end adaptor ligated-cDNA tags at the 3′ end were amplified using PCR indexed primers (IP) annealing in the adaptor sequence for 15 cycles enriching the final libraries (see Table S1 for all 6-nt tags/index). Libraries one through fifteen were indexed with non-HPLC purified IP 1–15 and the remaining fifteen libraries (technical replicates) were indexed with HPLC purified IP 16–30 utilizing the same cDNA sample (i.e., cDNA library 1 with IP 1 and IP 16).

The standard desalted non-HPLC primers (NH) primers were ordered in a 96 well plate (Integrated DNA Technologies, Coralville, Iowa, USA) designed with two empty wells between every well containing primer to allow the dispensing needle to be rinsed out twice before making a new primer. The HPLC purified primers (HP) were ordered individually (Integrated DNA Technologies, Coralville, Iowa, USA). All double stranded cDNA libraries had expected size (∼250 bp) when run on a 2% agarose gel except library 5 (third bioreplicate from control at time point 06 h) indexed with NH primer that failed (Table S1). The remaining 29 libraries were pooled together (20 ng/library), purified with 80% ethanol, concentrated with XP beads (Beckman Coulter, USA) and sent to the Core Laboratories Center Genomics, Institute of Biotechnology, Cornell University (http://www.biotech.cornell.edu/biotechnology-resource-center-brc) for paired-end sequencing (2×100 cycles + 7 cycle index read) performed with the HiSeq 2000 Illumina with ‘TruSeq PE Cluster Kit v3’ for the flow-cell and ‘TruSeq SBS kit v3’ for the sequencing reagents. The sequencing was performed in a single lane to minimize lane-to-lane variability between the technical replicates and rule out any lane-primer effects.

### Bioinformatics analysis – reads processing

A thorough quality control on the raw data was performed using FastQC software written in Java to provide summary statistics for FASTQ files (http://www.bioinformatics.babraham.ac.uk/projects/fastqc/) [22] and to report problems, thus ensuring the detection of biases in the data. For all the 29 libraries the phred-like quality scores (Qscores) was >20. The detection of sequencing adapters and primers, poor quality at the ends of reads, limited skewing at the ends of reads and N's were then processed and filtered out with the Ea-Utils software (http://code.google.com/p/ea-utils/wiki/FastqMcf) [23] increasing the Qscore to >30 for all the libraries and length >50 bp (Q30L50).

### *De novo* assembly

*De novo* assembly was performed with several assemblers for comparison purposes. Assembly was based on the de Bruijn graph [24] and included Trinity software with default settings (http://trinityrnaseq.sourceforge.net/) [9], Trans-ABySS with multi-k-assembled (http://www.bcgsc.ca/platform/bioinfo/software/trans-abyss) [10] and SOAPdenovo-trans with adjusted k-mers (http://soap.genomics.org.cn/SOAPdenovo-Trans.html) [11]. For all the *de novo* assemblies we used a server with 512 GB (Gigabytes) of RAM, 64 cores (CPUs) and CentOS as operating system.

In order to assess the quality of each assembly we compared the major outcomes: contig mean size, number of sequences (N50) and length (L50). We also compared the mean size distribution of assembled transcripts with ITAG2.3 tomato gene models [25]. All plots were generated using free and open-source ‘R software’ (R Development Core Team, 2010; http://www.R-project.org).

### Mapping and error estimation

All the reads from both technical replicates (non-HPLC and HPLC) were separately mapped against a Trinity HP *de novo* assembly using ‘Bowtie2’ (http://bowtie-bio.sourceforge.net/bowtie2/index.shtml) to screen for total error number and errors per read. The error percentage was calculated with the ‘Error Correction Evaluation Toolkit software’ [26] as (Error Number/Mapped Bases) ×100 and mapping percentage as (Total Reads/Mapped Reads)/Total Reads ×100 against a Trinity HP reference.

Since no significant differences were found with regards to mean error per read as expected, a final *de novo* assembly was performed with all the reads combined to increase the coverage of the transcripts, building a final reference using Trinity with default settings.

### Gene expression and differentially expressed genes

Gene expression was carried out with ‘RNA-Seq by Expectation-Maximization (RSEM)’ software (http://deweylab.biostat.wisc.edu/rsem/README.html) [27] bundled with the Trinity package. Differentially expressed transcripts across the time points for both control and salt-treated plants were identified and clustered according to expression profiles using ‘EdgeR Bioconductor’ package

(http://www.bioconductor.org/packages/2.11/bioc/html/edgeR.html) [28] using ‘R statistical software’ (R Development Core Team, 2010; http://www.R-project.org).

### Functional annotation

Functional annotation and Gene Ontology (GO) analysis was carried out using free and open source ‘Blast2GO’ software (http://www.blast2go.com/b2ghome) [29].

### Statistical analysis

Multivariate comparisons of transcriptional expression profiles between HP and NH samples were conducted using ‘R statistical software’ (R Development Core Team, 2010; http://www.R-project.org) including a permutational multivariate analysis of variance (ADONIS) with a Bray-Curtis distance matrix in the Vegan package. Fixed effects in the model included primer type, time point, and interactions.

## Results and Discussion

### Validation of technical replicates

Many RNA-seq experiments include both biological (RNA from different samples) and technical (same source of RNA) replicates [28]. In our work, technical replicates corresponded to transcript isoforms barcoded with both non-HPLC (NH) and HPLC (HP) purified index primers. Prior to data analysis, we evaluated if library construction with these two types of oligonucleotides resulted in significant differences by separately analyzing and comparing the output of both datasets (NH vs. HP) using different bioinformatics statistical analyses. Variance partitioning through permutational multiple analysis of variance indicates that the primer-choice (NH vs. HP) in the statistical model explained less than 2% of the variation in expression profiles whereas the overall model explained greater than 85% (Table S2A–E). The specific effect of primer-choice varied with the cut-off of the most expressed transcripts at 10, 100, 1,000, 10,000, and 100,000 RPKM (*P*-value  = 0.310, *P*-value  = 0.066, *P*-value  = 0.049, *P*-value  = 0.055, and *P*-value = 0.038, respectively). It should be noted that low significance in expression profiles (Table S2B-E) might be due to experimental and biological noise, rather than technical effects of primer purification. Slight variation between technical replicates without affecting datasets has also been found and described Marioni *et al.* (2008) [30]. A dendrogram of differentially expressed transcripts was created to visualize the relationship between technical and biological replicates, showing that the difference between technical replicates is smaller than biological replicates (Fig. S1). Lower variability in technical replicates than biological replicates is in accordance with Robinson *et al.* (2010) [28]. These findings validate our technical replicates, increase the robustness and accuracy of the transcriptome (i.e., more depth in the *de novo* assembled transcripts from both biological and technical replicates) and suggests that the use of NH index primers can be adopted, greatly reducing the cost of indexing step for future RNA-seq experiments. Even in the case that one library fails due to the use of non-HPLC primer (low probability, 6% in our case) it is still worth building libraries with cheaper primers, as the quality and quantity of data is not affected. Moreover, a failed library can be easily detected at early stages of library construction and thus barcoded with a new index primer and checked for expected size on an agarose gel (see Library preparation and sequencing – Material and Methods).

### Reads processing

The high-throughput and powerful RNA-seq technology has allowed scientists to reconstruct a transcriptome from species with no genomics information available, recovering most of the expressed genes in a given cell or tissue. For example, 454 GS FLX Titanium pyrosequencing has been used in olive tree (*Olea europaea*) [31] and the Illumina Genome Analyzer in Chinese cabbage (*Brassica rapa*) [32]. To do this, a suggested number of reads (>30 million pair-end reads >30 nucleotides for experiments whose purpose is to compare transcriptional profiles) should be generated either with 454 or Illumina platform to produce a meaningful assembled transcriptome [33]. One lane in an Illumina HiSeq2000 flow-cell will generate more than 100 million reads. To obtain a global view of the transcriptome of *Petunia x hybrida* from both control and salt-treated leaf samples we generated 196 million reads per lane (raw data) ranging from 10 to 23 million reads across the 29 libraries (Table S1), in accordance with the yield suggested by Goldfeder *et al*. (2011) [34].

### Transcriptome *de novo* assembly and evaluation

**Figure 1:**
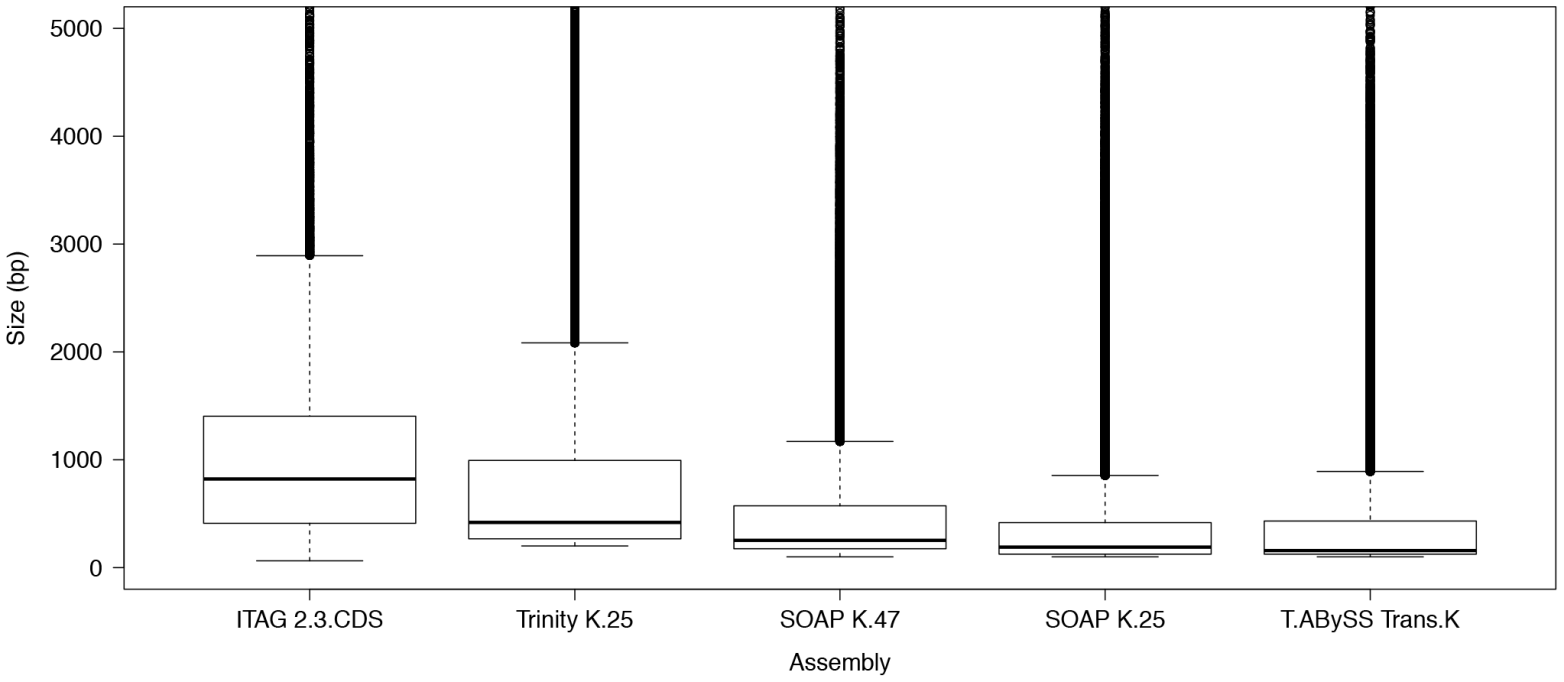
Boxplot comparisons of *de novo* assembled transcripts length distribution using Trinity, SOAPdenovo-trans and TransABySS software. First column (ITAG2.3 CDS) indicates tomato full CDS transcriptome, 2^nd^ column represents Trinity assembly using default k-mer set at 25. Third and 4^th^ columns represent assembly generated with SOAPdenovo-trans (SOAP) with k-mers (K) set at 25 and 47, respectively. Last column represents assembly generated with Trans-ABySS (T.ABySS) using trans k-mer. Transcripts longer than 5,000 bp were not plotted.

Comparison of software used in our study showed that Trinity outperformed the rest (Trans-ABySS and SOAPdenovo-trans) across the entire range of conditions and that Trans-ABySS had the lowest of the quality assembly (Fig.1). K-mer length was adjusted to include every odd numbers from 23 to 63 (i.e., k-mers 23, 25, …, up to 63) for Trans-ABySS (T.ABySS hereafter) and SOAPdenovo-trans (SOAP hereafter) to optimize transcriptome *de novo* assembly into contigs and scaffolds. The best results with SOAP were obtained with k-mer length 47, which yielded larger contigs and scaffolds (data not shown), that had higher N50 and L50 than other k-mer lengths (Table 1). T.ABySS yielded longer contigs using ‘trans k-mer’. The best results were obtained with Trinity *de novo* assembler (default k-mer 25) recovering more full-length transcripts across all the samples and all expression levels. This result is similar of those presented in the studies by Grabherr *et al*. (2011) [9] and Xu *et al*. (2012) [35]. However, this result is not in accordance with the finding of Vijay *et al.* (2013) [36]. In their results SOAP outperformed all three assemblers (T.ABySS, SOAP and Trinity). This shows the importance of optimizing a methodology for a particular dataset, as all datasets are different. Summary of results including contig mean size, N50 and L50 for all the assemblers are found in Table 1.

**Table 1:** Summary of results from *de novo* assembly with Trinity, SOAPdenovo-trans and Trans-ABySS software.

	Contig		
Software/K-mer (K.)	MS	L.50	N.50
Trinity/K.25	822	1,505	22,452
SOAPdenovo-trans/K.47	449	720	20,142
SOAPdenovo-trans/K.25	342	510	31,210
Trans-ABySS/trans K.	392	851	36,849

Trinity outperformed all assemblers with default k-mer. The best result with SOAPdenovo-trans was obtained with k-mer length 47 and T.ABySS yielded longer contigs using ‘trans k-mer’.

MS =  Mean size (bp).

L50 = Minimum contig length (bp) representing 50% of the assembly.

N50  =  Minimum number of contigs representing 50% of the assembly.

To evaluate sequence length of the recovered *Petunia* transcriptome, we compared the apparent total mRNA length to the fully annotated tomato transcriptome. Tomato was utilized as the most closely related species (both in family Solanaceae) with a full-annotated transcriptome (34,727 CDS, N50 7,000 sequences with 1,400 bp average length) [25]. The comparison was made using the three aforementioned assemblers looking at mRNA size distribution; we observed that Trinity showed the closest distribution to tomato transcriptome followed by SOAP k-mer 47 and lastly by T.ABySS trans k-mer (Fig. 1). Thus, according to our data, Trinity is the most accurate assembler leading to a transcript mean size closer to tomato's.

### Transcriptome functional annotation

Our final proposed reference transcriptome has a size of 111 MB, in which we have identified 101,447 unigenes with 135,814 isoform/transcript fragments. Basic Local Alignment Search Tool (BLAST) indicates that 32% (32,879 unigenes) from the total number of unigenes in the transcriptome map directly to *Solanum lycopersicum* coding DNA sequence (CDS) with a sequence average size of 997 bp, 0.04% (40 unigenes) map to plant ribosomal proteins with an average size of 445 bp and 2% (2,148 unigenes) map to bacterial genes with an average size of 377 bp. The remaining sequences (65% of the dataset, 66,380 unigenes) do not show any similarity with these protein datasets. The high number of unmapped unigenes may be accounted for the variable regions not represented in the set used for BLAST (i.e., a minority of variables UTR sites in *Petunia* genes do not resemble *Solanum lycopersicum* sequences and transposable element sequences specific to *Petunia*). Overall, we found that the quality of the predicted *Petunia* genes was comparable to the well-annotated tomato genome. Huang *et al*. (2012) generated ∼192 millions Illumina reads sequencing roots and leaves from *Milletia pinnata* (Semi-Mangrove), growing under fresh and seawater (∼500 mM NaCl), which were assembled into 108,598 unigenes [37]. Of these, 50.3% (54,596) showed significant similarities with protein databases and 1% were annotated with sequences from non-plant sources. The three species with the most BLAST hits in our work were *Vitis vinifera*, *Solanum lycopersicum* and *Glycine max.* A graph with species distribution and their BLAST hits is found in Fig. S2.

**Figure 2:**
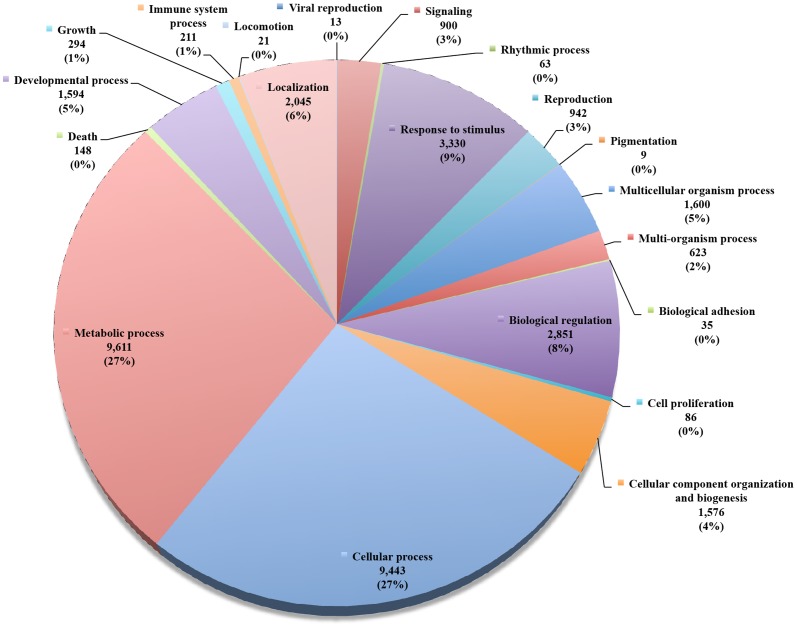
Gene Ontology analysis in the *Petunia x hybrida* reference transcriptome assembled with Trinity software.

Gene Ontology (GO) was used to classify functions of the assembled transcripts, from which we obtained a total number of 69,277 GO term annotations in our proposed transcriptome. The large majority of unigenes corresponded to metabolic process (9,611), cellular process (9,443) and response to stimulus (3,330) (Fig. 2). Transcriptome GO terms and gene descriptions are found in Table S3 and DNA sequences are deposited it in the SOL Genomics Network (SGN) database http://solgenomics.net for others to use. This all-reads-assembly performed with Trinity was used for further analysis. In our work we used Bowtie mapper bound with the Trinity package, which mapped back to the final reference transcriptome ∼18 million reads (data not shown).

### Gene expression and differentially expressed genes

The top five most highly expressed transcripts (highest RPKM) were the same for each of the 29 libraries regardless of presence of salt stress. These five genes are involved in photosynthesis, as expected for leaf samples (Table 2). The most highly expressed gene (highest RPKM) across all the samples was the small chain of ribulose-bisphosphate carboxylase (EC 4.1.1.39). The high expression of rubisco corresponds with maize B73 seedlings exposed to low night temperature (4°C) as determined by real-time PCR [38]. Transcript abundance and functional annotation for the top five most expressed genes with their respective RPKM expression levels is shown in Table 2.

**Table 2:** Comparison and functional annotation of transcript abundance in ‘Reads per Kilobase of Exon per Million Reads Mapped’ (RPKM) and functional annotation of the 5 most expressed transcripts.

Seq. Name	RPKM	Seq. Description	Seq. Length (bp)	eValue
comp27110_c0	134,428±6,490	Ribulose-bisphosphate carboxylase (EC 4.1.1.39) small chain - petunia CAA27444.1	795	4.00E-178
comp28131_c1	45,112±2,420	Petunia gene for chlorophyll a/b binding protein cab 25	1,184	0.00E+00
comp28218_c0	29,073±2,192	Ribulose-bisphosphate carboxylase (EC 4.1.1.39) small chain - petunia CAA27445.1	1,565	9.00E-128
comp28216_c0	27,562±1,932	Photosystem I reaction center II	1,564	3.00E-137
comp25306_c0	12,007±451	Chlorophyll a-b binding protein chloroplastic-like	1,602	7.46E-144

The first two columns transcript abundance measured in RPKM (Avg ± S.E) for the top five most expressed genes across the 29 libraries. Third column is sequence (Seq.) description obtained through functional annotation used in Blast2GO software. Sequence length of *de novo* assembled transcripts varied for all the transcripts shown.

#### Differentially expressed genes

When comparing the total number of differentially expressed genes and transcripts across the three time points in a pair-wise fashion, we observed that differential expression was higher in salt treated plants compared to a control at a particular time point. For example, the large majority of differentially expressed genes (1,064) and transcripts (1,494) were found between salt treated plants at 24 h vs. control at 06 h (Table 3). The number of genes differentially expressed in the control (00 h, 06 h and 24 h) is likely due to transcripts involved in plant circadian rhythm and mechanical damage induced while sampling.

**Table 3:** Pair-wise matrix comparison of differentially expressed transcripts and genes (genes in parenthesis) of leaves exposed to 0 and 150 mM NaCl across three different times (0, 6 and 24 h).

	CTR_00h	CTR_06h	CTR_24h	STR_06h	STR_24h
CTR_00h	0 (0)	885 (718)	237 (186)	1,058 (790)	710 (502)
CTR_06h	.	0 (0)	526 (440)	905 (669)	1,494 (1,064)
CTR_24h	.	.	0 (0)	780 (553)	882 (644)
STR_06h	.	.	.	0 (0)	174 (143)
STR_24h	.	.	.	.	0 (0)

CTR = Control, STR =  150 mM NaCl,

_00 = 0 h after NaCl; _06 = 6 h after NaCl; _24 =  24 h after NaCl.

**Figure 3:**
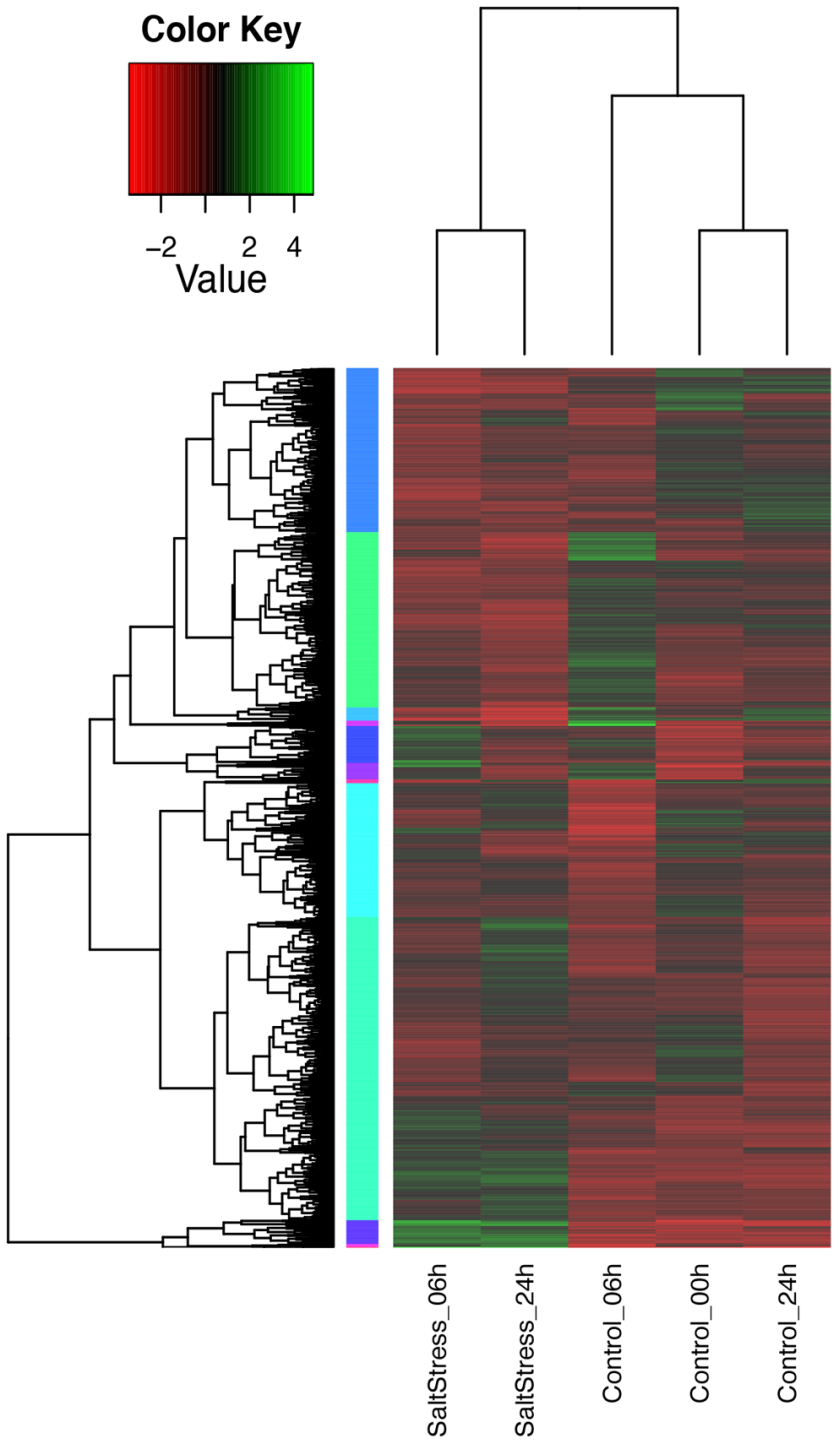
Heatmap of differentially expressed transcript isoforms across the three time points. Green and red colors indicate up- and down- regulated transcripts, respectively, from both control and salt treated leaves. False Discovery Rate (FDR) ≤0.001 and the maximum value of |log_2_ (ratio of stress/control)| ≥1 was used as cut-off to evaluate significant differences in expression.

**Figure 4:**
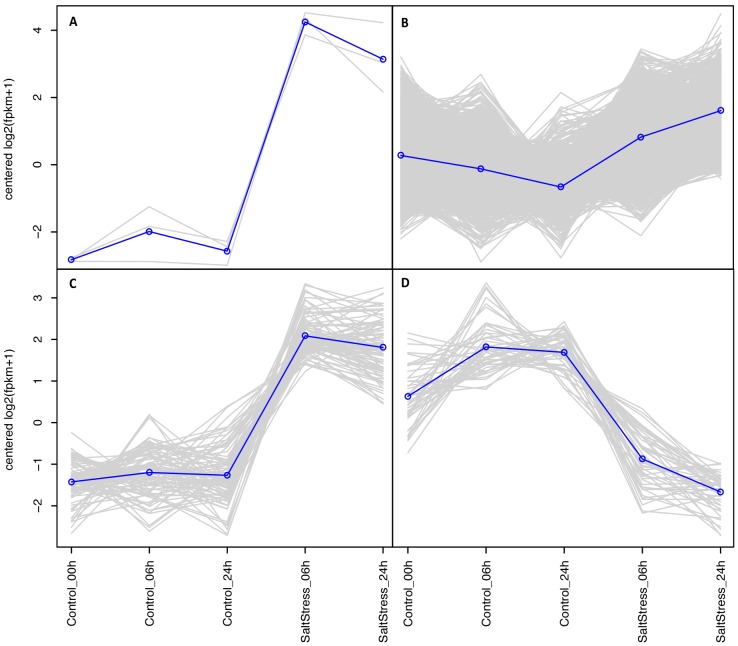
Subclusters with differently up- and down- regulated transcript isoforms. In all panels (A–D) gray color lines indicates individual transcript expression levels and blue line indicates a ‘consensus’ of all the transcripts within a specific subcluster. (A) Corresponds to subcluster 12 with 3 up-regulated transcripts, (B) corresponds to subcluster 2 with 1,125 up-regulated transcripts, and (C) corresponds to subcluster 7 with 88 up-regulated transcripts. (D) Corresponds to subcluster 4 with 49 down-regulated transcripts.

To represent differentially expressed genes under salt stress we created a heatmap of RPKM-normalized transcript isoforms through hierarchical clustering. False Discovery Rate (FDR) ≤0.001 and the maximum value of |log_2_ (ratio of stress/control)| ≥1 was used as cut-off to evaluate significant differences in expression (Fig. 3). We found 1,216 up-regulated transcripts (grouped in 3 subclusters) and 49 down-regulated transcripts (grouped in 1 subcluster) whose expressions were significantly induced and reduced by NaCl treatment, respectively (Fig. 4). Three isoforms of heat shock protein (HSP) were the most up-regulated transcripts, increasing their expression by over 90-fold (Fig. 4A). The high expression level of HSP under abiotic stress is in accordance with the DNA microarray analysis in *Arabidopsis* by Seki *et al.* (2002) [39]. The large majority of up-regulated transcripts (1,125) increased their expression between 2 and 50-fold after 06 h and 24 h of stress (Fig. 4B). These transcripts were involved in phosphorylation processes (i.e., serine threonine-protein kinase edr1-like and serine threonine-protein kinase NAK) and motor proteins (i.e., kinesin-like protein kin12b-like and myosin-like protein), to name a few. These findings are similar to those reported by Yu *et al.* (2011) in their transcriptome profile of dehydration stress in the Chinese cabbage [33]. Transcripts involved in vesicle trafficking and cytoskeletal dynamics were also found in this subcluster. The results of Mazel *et al.* (2004) support that vesicle trafficking plays an important role in plant adaptation to stress [40]. Transgenic plants expressing the *Arabidopsis RabG3* (vesicle trafficking-regulating gene) under the constitutive 35S promoter increased tolerance to salt in transgenic plants, accumulating more sodium in the vacuoles. Interestingly, many transcripts in this cluster were also involved in plant disease resistance (i.e., late blight resistance protein homolog r1a-10-like and disease resistance protein R3a-like MYB protein) suggesting a crosstalk response with biotic stress. AbuQamar *et al.* (2009) reported that the *R2R3MYB* transcription factor is induced by pathogens, plant hormones and salinity in *Solanum lycopersicum* [41]. Eighty-eight up-regulated transcripts increased their expression by 30 to 50-fold (Fig. 4C) and only 49 transcripts were down-regulated (Fig. 4D).

In contrast to NaCl treatment, most of the up- and down- regulated transcripts between control treatments were involved in oxidation-reduction processes, photosynthetic electron transport in photosystem II, electron carrier activity, response to cyclopentenone, coenzyme binding, cytochrome P450 regulation and transferase activity. A detailed lists with all up-regulated transcripts (subclusters 12, 2 and 7) and down-regulated transcripts (subcluster 4), including gene descriptions, changes in expression and their GO annotation are found in Table S4A–D.

#### Candidate genes to enhance salt tolerance

**Figure 5:**
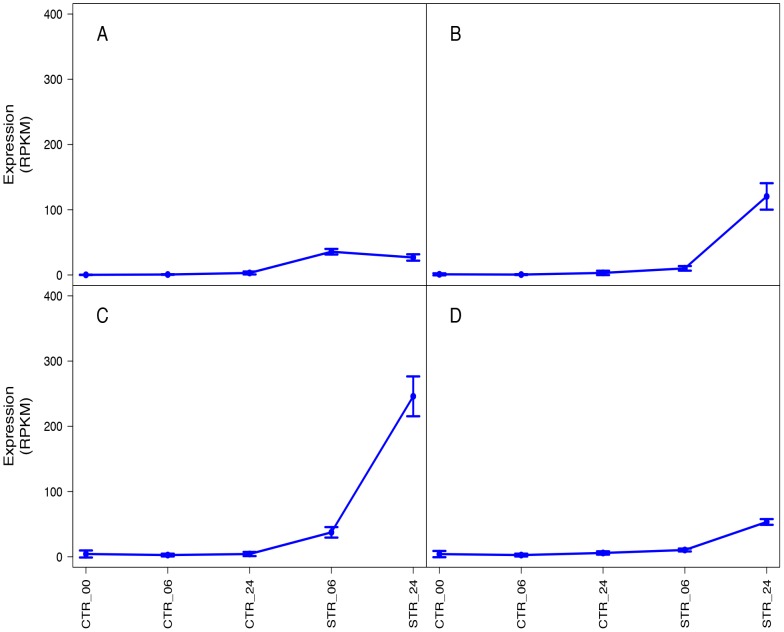
Candidate genes selected based on their high induction levels (RPKM). Candidate genes induced at both 06 and 24 h of salt stress are plotted in four panels; (A) Oleosin Bn−V−like, (B) Homeobox-leucine zipper protein ATHB-7-like, (C) Unknown (D) Putative ribonuclease H protein At1g65750-like.

**Figure 6:**
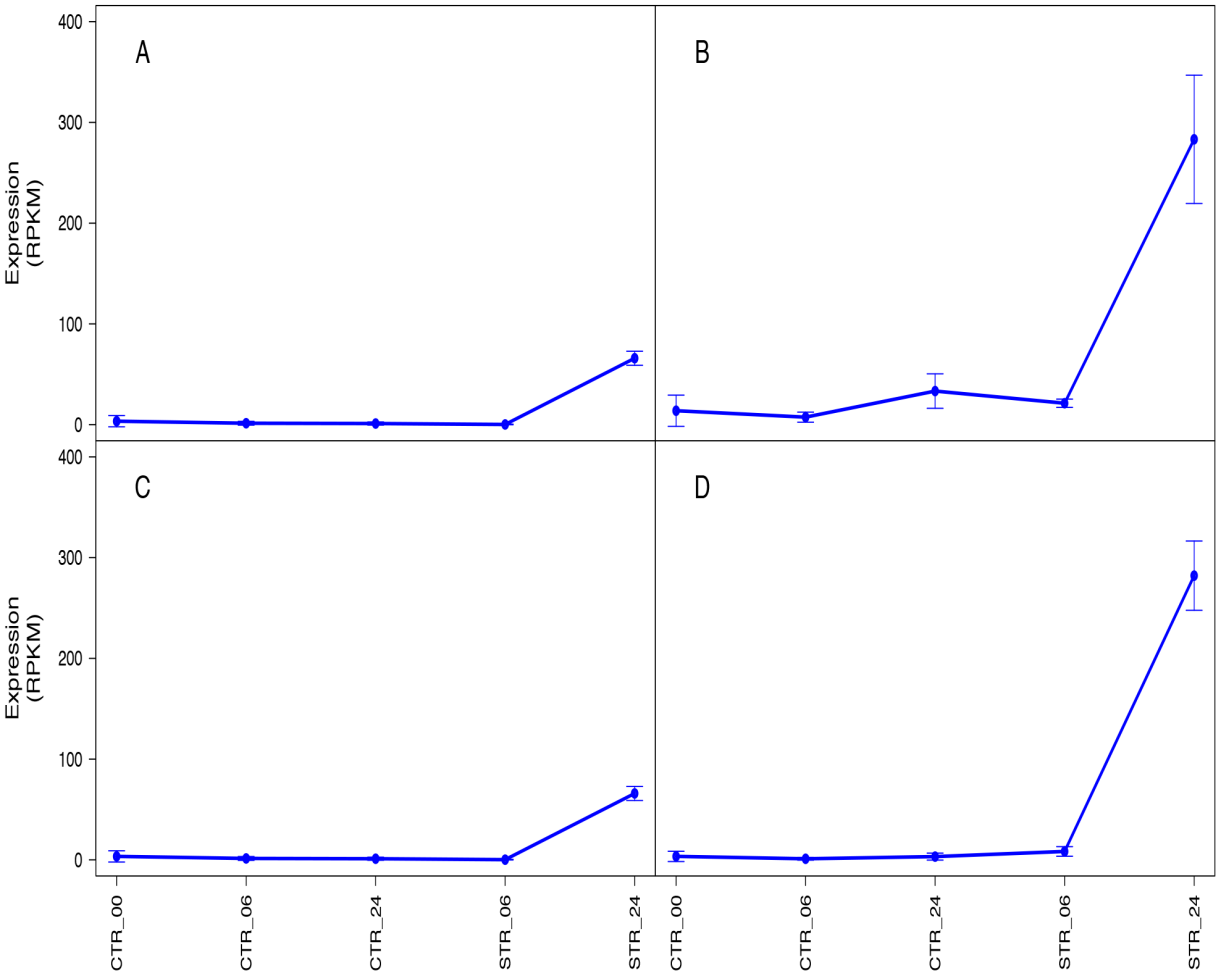
Candidate genes selected based on their high induction levels (RPKM). Candidate gens induced at 24 h of salt stress but not at 6 h are plotted in four panels; (A) Expansin-like B1-like, (B) Bidirectional sugar transporter SWEET11-like, (C) Phosphoenolpyruvate carboxylase kinase and (D) Low-temperature-induced 65 kDa protein-like.

Based on our analysis we suggest eight salt-induced genes that could be further studied. Functional analysis for these candidate genes may be useful for genetic engineering or marker assisted selection to enhance salt tolerance in Solanaceae. We group the candidate genes into two major groups; those induced at both 06 and 24 h of salt stress (Fig. 5) and those induced at 24 h of stress but not with 6 h (Fig. 6). From the eight suggested candidate genes, no homology (unknown protein) was retrieved upon performing BLASTX to the tomato genome (ITAG release 2.31), with ‘comp32475_c0_seq1’. The ‘unknown’ transcript maps to tomato chromosome 3 between 61,095,606-61,097,016 base pairs and it is induced 17-fold when comparing control 06 h vs. salt 06 h and 59 fold when comparing control 06 h vs. salt 24 h. Genes ID, annotation, *P*-values, FDR and fold induction for the suggested candidate genes are shown in Table 4. Partial DNA sequences can be found at the SOL Genomics Network (SGN).

**Table 4:** List of eight salt-induced candidate genes at both 06 and 24 h of salt stress and at 24 h of salt stress alone.

						Induced	
ID	Description	logFC	logCPM	PValue	FDR	Salt 06 h	Salt 24 h
comp45963_c0_seq1	Oleosin Bn-V-like	5.7472	4.1995	8.28E-35	9.91E-31	32-Fold	27-Fold
comp32475_c0_seq1	Unknown	3.6723	5.6885	4.48E-47	1.31E-44	17-Fold	59-Fold
comp32085_c0_seq1	Homeobox-leucine zipper protein ATHB-7-like	3.4728	3.9414	1.30E-20	1.48E-17	14-Fold	42-Fold
comp32085_c0_seq1	Putative ribonuclease H protein At1g65750-like	3.4728	3.9414	1.30E-20	1.48E-17	4-Fold	19-Fold
comp14467_c0_seq2	Phosphoenolpyruvate carboxylase kinase	4.8604	4.8598	2.16E-39	4.23E-37	.	45-Fold
comp31034_c0_seq1	Low-temperature-induced 65 kDa protein-like	2.9372	3.4215	3.41E-07	2.24E-05	.	41-Fold
comp40589_c0_seq1	Expansin-like B1-like	10.7912	5.2361	3.00E-64	1.70E-61	.	35-Fold
comp26249_c0_seq5	Bidirectional sugar transporter SWEET11-like	3.6832	7.2841	7.29E-54	2.81E-51	.	28-Fold

Gene's ID and description are represented in the first two columns and DNA sequences for all the transcripts are found in the SOL Genomics Network (SGN) database. Induction (Fold) upon salt stress is listed in the last two columns.

#### Gene Ontology analysis

To better characterize the effects of NaCl in biological processes we conducted GO enrichment analysis using Fisher's Exact Test (Bonferroni-corrected, FDR ≤0.05), with differentially expressed genes and the whole transcritpome set as a background reference. With the exception of ‘regulation of biological quality’, all the statistically significant overrepresented GO terms in salt treated leaves from 6 h were the same as those from 24 h. The most overrepresented GO terms in response to NaCl stress were ‘response to abscisic acid stimulus’, ‘response to jasmonic acid stimulus’, ‘response to ethylene stimulus’, ‘response to salt stress’ and ‘G-protein coupled photoreceptor activity’, indicating that most induced genes at this early stage of the stress are not salt-induced but genes involved with osmotic adjustment, hormonal changes and stress signaling (Table S5A–C). These results are in accordance with previous reports on salt stress studies [41]. More interestingly, 72 significantly enriched GO terms were associated exclusively with samples at 24 h of salts stress (i.e., not found at 6 h). From these results, we observe that salt induces the activation of a distinct group of genes not activated previously, suggesting that the concentration of Na^+^ or Cl^−^ ions may interfere with cellular functions and biological processes such as the DNA replication process (i.e. GO terms: ‘DNA replication’, DNA conformation change’, DNA replication initiation’, ‘DNA-dependent DNA replication’), metabolic processes (‘nucleic acid metabolic process’, ‘glycerolipid metabolic process’, ‘RNA metabolic process’), transport (‘nuclear transport’, ‘oligopeptide transmembrane transport’, ‘nucleocytoplasmic transport’, ‘nitrogen compound transport’) and development (‘post-embryonic development’, ‘developmental process’). The 72 GO terms are listed in Table 5. Ulm (2004) reported that Na^+^ accumulation may also cause genotoxicity in which DNA alteration/damage can arise as a consequence of errors in DNA replication and DNA repair [42]; Katsuhara and Kawasaki (1996) showed nuclear deformation and genotoxicity in the meristematic root cells of barley (*Hordeum vulgare*) in salt-treated plants grown hydroponically under 200 mM NaCl. In their experiment, cells showed deformed and degraded nuclei after 4 h of salt stress whereas untreated cells showed nuclei with smooth and clear boundaries [43]. This suggests that the genotoxicity effects of NaCl may affect grasses faster than Solanaceous plants. A complete list of all the GO terms and their respective unigenes at time point 0 h and are fond in Table S5A. An enriched Gene Ontology analysis through Fisher's exact test with multiple testing correction of FDR for control and salt treated samples at time points 06 h and 24 h are found in Tables S5B–C. DNA sequences corresponding to specific unigenes associated with GO terms can be found in the SOL Genomics Network (SGN) http://solgenomics.net webpage.

**Table 5:** Unique Gene Ontology (GO) terms associated with samples at 24 h after salt stress.

GO-ID	Term	Category	FDR
GO:1901618	organic hydroxy compound transmembrane transporter activity	F	5.30E-11
GO:1901576	organic substance biosynthetic process	P	8.90E-03
GO:1901476	carbohydrate transporter activity	F	4.33E-04
GO:0090304	nucleic acid metabolic process	P	2.18E-03
GO:0080029	cellular response to boron-containing substance levels	P	5.30E-11
GO:0071918	urea transmembrane transport	P	5.30E-11
GO:0071705	nitrogen compound transport	P	1.86E-03
GO:0071702	organic substance transport	P	1.65E-02
GO:0071496	cellular response to external stimulus	P	2.23E-02
GO:0071103	DNA conformation change	P	2.91E-02
GO:0051649	establishment of localization in cell	P	3.19E-02
GO:0051640	organelle localization	P	1.33E-05
GO:0051169	nuclear transport	P	1.07E-02
GO:0051168	nuclear export	P	1.43E-02
GO:0046715	borate transmembrane transporter activity	F	5.30E-11
GO:0046713	borate transport	P	5.30E-11
GO:0046486	glycerolipid metabolic process	P	2.40E-02
GO:0044255	cellular lipid metabolic process	P	1.48E-02
GO:0043566	structure-specific DNA binding	F	5.54E-05
GO:0042887	amide transmembrane transporter activity	F	6.00E-09
GO:0042886	amide transport	P	1.67E-05
GO:0035673	oligopeptide transmembrane transporter activity	F	1.54E-02
GO:0035672	oligopeptide transmembrane transport	P	1.54E-02
GO:0035445	borate transmembrane transport	P	5.30E-11
GO:0035384	thioester biosynthetic process	P	3.75E-02
GO:0034660	ncRNA metabolic process	P	1.58E-03
GO:0032502	developmental process	P	2.75E-02
GO:0032501	multicellular organismal process	P	4.93E-02
GO:0031669	cellular response to nutrient levels	P	9.35E-03
GO:0031668	cellular response to extracellular stimulus	P	2.23E-02
GO:0031667	response to nutrient levels	P	1.66E-02
GO:0019755	one-carbon compound transport	P	5.30E-11
GO:0016099	monoterpenoid biosynthetic process	P	1.07E-02
GO:0016098	monoterpenoid metabolic process	P	1.07E-02
GO:0016070	RNA metabolic process	P	6.06E-03
GO:0015850	organic hydroxy compound transport	P	6.85E-11
GO:0015840	urea transport	P	5.30E-11
GO:0015793	glycerol transport	P	2.15E-10
GO:0015791	polyol transport	P	1.63E-11
GO:0015665	alcohol transmembrane transporter activity	F	1.63E-11
GO:0015440	peptide-transporting ATPase activity	F	1.54E-02
GO:0015421	oligopeptide-transporting ATPase activity	F	1.54E-02
GO:0015204	urea transmembrane transporter activity	F	7.31E-10
GO:0015168	glycerol transmembrane transporter activity	F	2.15E-10
GO:0015166	polyol transmembrane transporter activity	F	1.63E-11
GO:0015144	carbohydrate transmembrane transporter activity	F	4.33E-04
GO:0015103	inorganic anion transmembrane transporter activity	F	8.14E-04
GO:0010157	response to chlorate	P	1.54E-02
GO:0010036	response to boron-containing substance	P	5.30E-11
GO:0010027	thylakoid membrane organization	P	1.12E-03
GO:0009991	response to extracellular stimulus	P	3.75E-02
GO:0009791	post-embryonic development	P	4.79E-02
GO:0009704	de-etiolation	P	3.75E-02
GO:0009668	plastid membrane organization	P	1.12E-03
GO:0009658	chloroplast organization	P	1.88E-06
GO:0009657	plastid organization	P	3.78E-05
GO:0009605	response to external stimulus	P	1.64E-02
GO:0009536	plastid	C	3.19E-02
GO:0009507	chloroplast	C	2.27E-02
GO:0009058	biosynthetic process	P	4.29E-02
GO:0008765	UDP-N-acetylmuramoylalanyl-D-glutamate-2,6-diaminopimelate ligase	F	1.54E-02
GO:0008610	lipid biosynthetic process	P	4.05E-05
GO:0008509	anion transmembrane transporter activity	F	2.34E-02
GO:0006996	organelle organization	P	2.07E-02
GO:0006913	nucleocytoplasmic transport	P	1.07E-02
GO:0006820	anion transport	P	3.97E-02
GO:0006650	glycerophospholipid metabolic process	P	1.21E-02
GO:0006270	DNA replication initiation	P	1.43E-02
GO:0006261	DNA-dependent DNA replication	P	1.84E-04
GO:0006260	DNA replication	P	1.72E-02
GO:0003677	DNA binding	F	4.93E-02
GO:0003676	nucleic acid binding	F	1.94E-02

False Discovery Rate (FDR) cut-off was set at 0.05, and all biological GO terms were significantly overrepresented.

The compartmentalization of Na^+^ into the vacuole by the Na^+^/H^+^ tonoplast antiporter is a mechanism employed by some plants to cope with salt [44]–[46]. Tomato plants overexpressing an *Arabidopsis* vacuolar Na^+^/H^+^ antiporter (*AtNHX1*) were able to grow in the presence of 200 mM sodium chloride accumulating high sodium concentrations in leaves but not in fruits [46]. However, we did not observe this mechanism in our experiment. We believe that after 24 h of salt stress, while initial cellular damage can be evident, a longer-term response may be required to observe genes involved in exclusion and/or compartmentalization of ions. Future work with RNA-seq should seek to understand the longer-term detrimental consequences of salt in in Solanaceae plants.

In this work we carried out the first in-depth transcriptomic analysis in *Petunia* under salt stress through RNA-seq. We quantified the expression of more than 7,000 genes across 24 h of acute NaCl stress. The large number of up- and down- regulated transcripts in response to salt stress is consistent with previous research and the underlying physiological responses to NaCl treatment. Stress response genes related to reactive oxygen species, transport, and signal transductions as well as novel and undescribed genes were identified. The candidate genes identified in this study can be applied as markers for breeding efforts or as candidates to genetically engineer plants to enhance salt tolerance. GO terms analyses indicated that most of the NaCl damage happened at 24 h inducing genotoxicity, affecting transport and organelles due to the high concentration of Na^+^ ions. We suggest that future RNA-seq with members of the Solanaceae incorporate more time points (i.e., longer exposure to NaCl) to assess detrimental effects of sodium chloride in plants. In this work we also propose a novel *Petunia* transcriptome assembled out of 196 million Illumina reads with Trinity software that can be used as an excellent tool for biological and bioinformatic inferences in the absence of an available genome. Additionally, we introduced a slight modification in the library preparation barcoding samples with non-HPLC primers. The methodological improvement presented could benefit the work in different next generation sequencing technologies, where the use of HPLC purified primers is an important contribution to the cost of sample preparation, thereby reducing a barrier to researchers of limited means to use high-throughput RNA sequencing.

## Supporting Information

Figure S1 Clustering of differentially expressed transcripts when comparing dispersion between biological (r1, r2, r3) vs. technical replicates (NH and HP). Conrtrol00h, Controlh06 and Control24h indicate control leaves samples taken at time 0 h, 6 h and 24 h respectively, after treatment commenced. SaltStress00h, SaltStress06h, SaltStress24h indicate salt treated leaf samples at the same time points. Clustering between NH and HP datasets is smaller for the biological replicates.
(TIFF)

Figure S2 Species distribution and their BLAST Hits.
(TIF)

Table S1 Million reads per library before (raw data, column 3) and after data cleaning (filtered data, column 4). The 6 unique nucleotides tags used to index each library are shown in the first column and yield (GB) per library in column 2. Note that library 5 indexed with non-HPLC primer failed.
(DOCX)

Table S2 **A.** Variance partitioning in Permutational Multiple Analysis of Variance (ADONIS) comparing expression profiles of the 10 most expressed transcripts between HPLC (HP) vs. non-HPLC (NH) datasets. B. Variance partitioning in Permutational Multiple Analysis of Variance (ADONIS) comparing expression profiles of the 100 most expressed transcripts between HPLC (HP) vs. non-HPLC (NH) datasets. C. Variance partitioning in Permutational Multiple Analysis of Variance (ADONIS) comparing expression profiles of the 1,000 most expressed transcripts between HPLC (HP) vs. non-HPLC (NH) datasets. D. Variance partitioning in Permutational Multiple Analysis of Variance (ADONIS) comparing expression profiles of the 10,000 most expressed transcripts between HPLC (HP) vs. non-HPLC (NH) datasets. E. Variance partitioning in Permutational Multiple Analysis of Variance (ADONIS) comparing expression profiles of the 100,000 most expressed transcripts between HPLC (HP) vs. non-HPLC (NH) datasets.
(DOCX)

Table S3 *Petunia x hybrida* cv. ‘Mitchell Diploid’ transcriptome and its functional annotation.
(XLSX)

Table S4 A. Up-regulated transcripts in cluster 12, changes in expression and GO terms annotation. B. Up-regulated transcripts in cluster 2, changes in expression and GO terms annotation. C. Up-regulated transcripts in cluster 7, changes in expression and GO terms annotation. D. Down-regulated transcripts in cluster 4, changes in expression and GO terms annotation.
(XLSX)

Table S5 A. Enriched Gene Ontology analysis and unigenes associated at CTR_00 through Fisher's exact test with multiple testing correction of False Discovery Rate (FDR). B. Enriched Gene Ontology analysis and unigenes associated between CTR_06 and STR_06 through Fisher's exact test with multiple testing correction of False Discovery Rate (FDR). C. Enriched Gene Ontology analysis and unigenes associated between CTR_24 and STR_24 through Fisher's.
(XLSX)
