# Correction: Transcriptomic Analysis of *Petunia hybrida* in Response to Salt Stress Using High Throughput RNA Sequencing

**DOI:** 10.1371/journal.pone.0099146

**Published:** 2014-05-27

**Authors:** The PLOS ONE Staff

## Notice of Republication

This article was republished on May 5, 2014, to correct the images and legends of Figures 5 and 6, and hyperlinked text in the Materials and Methods. Please download this article again to view the correct version. The originally published, uncorrected article and the republished, corrected article are provided here for reference.

## Supporting Information

File S1 Originally published, uncorrected article.
(PDF)

File S2 Republished, corrected article.
(PDF)
