# Supplementary figures and images for: Transcriptomic Analysis of Petunia hybrida in Response to Salt Stress Using High Throughput RNA Sequencing
Source: PLoS One. 2014 Apr 10;9(4):e94651. doi: 10.1371/journal.pone.0094651 (PMC3983219; doi:10.1371/journal.pone.0094651)

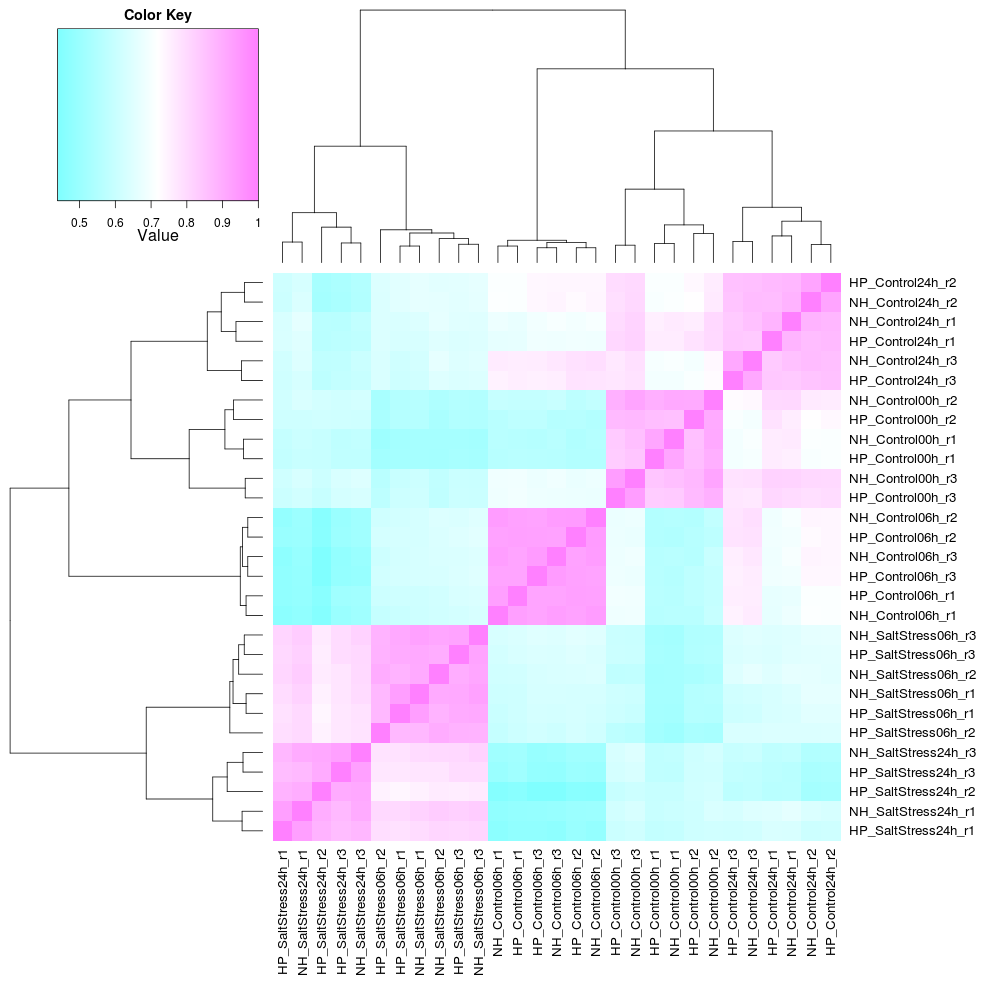

Supplement: Figure S1 — Clustering of differentially expressed transcripts when comparing dispersion between biological (r1, r2, r3) vs. technical replicates (NH and HP). Conrtrol00h, Controlh06 and Control24h indicate control leaves samples taken at time 0 h, 6 h and 24 h respectively, after treatment commenced. SaltStress00h, SaltStress06h, SaltStress24h indicate salt treated leaf samples at the same time points. Clustering between NH and HP datasets is smaller for the biological replicates. (TIFF) [file pone.0094651.s001.tif]

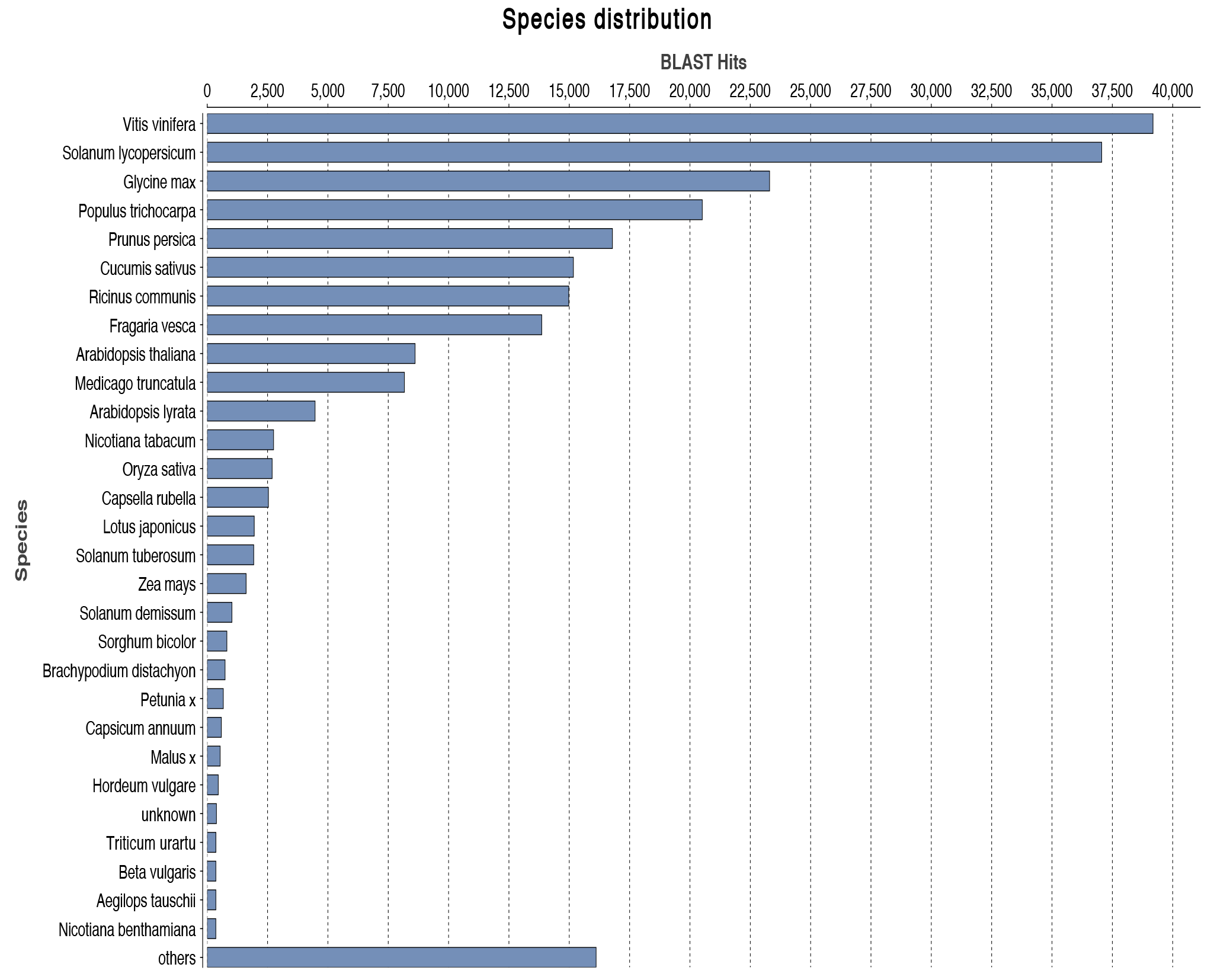

Supplement: Figure S2 — Species distribution and their BLAST Hits. (TIF) [file pone.0094651.s002.tif]
