# Supplementary material for: Transcriptomic Analysis of Petunia hybrida in Response to Salt Stress Using High Throughput RNA Sequencing
Source: PLoS One. 2014 Apr 10;9(4):e94651. doi: 10.1371/journal.pone.0094651 (PMC3983219; doi:10.1371/journal.pone.0094651)
Supplement: Table S1 — Million reads per library before (raw data, column 3) and after data cleaning (filtered data, column 4). The 6 unique nucleotides tags used to index each library are shown in the first column and yield (GB) per library in column 2. Note that library 5 indexed with non-HPLC primer failed. (DOCX) [file pone.0094651.s003.docx]

**Supporting Information**

Table S1.

| Library/primer type | Sample / Rep | Tag/Index | Yield (GB) | Row Reads |  |  | |  |
| --- | --- | --- | --- | --- | --- | --- | --- | --- |
| Lib. 1. Non-HPLC | Leaf CTR_00 h /R1 | ATCACG | 1,355 | 14,299,136 |  | |  | |
| Lib. 1. HPLC | Leaf CTR_00 h /R2 | CCGTCC | 1,045 | 10,962,764 |  | |  | |
| Lib. 2. Non-HPLC | Leaf CTR_00 h /R3 | CGATGT | 2,200 | 23,084,764 |  | |  | |
| Lib. 2. HPLC | Leaf CTR_00 h /R4 | GTAGAG | 982 | 10,319,494 |  | |  | |
| Lib. 3. Non-HPLC | Leaf CTR_00 h /R5 | TTAGGC | 1,724 | 18,140,264 |  | |  | |
| Lib. 3. HPLC | Leaf CTR_00 h /R6 | GTCCGC | 1,119 | 11,751,364 |  | |  | |
| Lib. 4. Non-HPLC | Leaf CTR_06 h /R1 | TGACCA | 2,085 | 21,904,108 |  | |  | |
| Lib. 4. HPLC | Leaf CTR_06 h /R2 | GTGAAA | 1,026 | 10,766,412 |  | |  | |
| Lib. 5. Non-HPLC | Leaf CTR_06 h /R3 | CACTGT | . | . |  | |  | |
| Lib. 5. HPLC | Leaf CTR_06 h /R4 | GTGGCC | 1,273 | 13,362,088 |  | |  | |
| Lib. 6. Non-HPLC | Leaf CTR_06 h /R5 | GCCAAT | 1,375 | 14,459,178 |  | |  | |
| Lib. 6. HPLC | Leaf CTR_06 h /R6 | GTTTCG | 987 | 10,365,066 |  | |  | |
| Lib. 7. Non-HPLC | Leaf STR_06 h /R1 | CAGATC | 1,595 | 16,771,012 |  | |  | |
| Lib. 7. HPLC | Leaf STR_06 h /R2 | CGTACG | 1,156 | 12,130,828 |  | |  | |
| Lib. 8. Non-HPLC | Leaf STR_06 h /R3 | ACTTGA | 1,566 | 16,458,846 |  | |  | |
| Lib. 8. HPLC | Leaf STR_06 h /R4 | GAGTGG | 952 | 9,981,366 |  | |  | |
| Lib. 9. Non-HPLC | Leaf STR_06 h /R5 | GATCAG | 1,742 | 18,340,684 |  | |  | |
| Lib. 9. HPLC | Leaf STR_06 h /R6 | GGTAGC | 1,171 | 12,287,296 |  | |  | |
| Lib. 10. Non-HPLC | Leaf CTR_24 h /R1 | TAGCTT | 1,211 | 12,733,646 |  | |  | |
| Lib. 10. HPLC | Leaf CTR_24 h /R2 | ACTGAT | 1,044 | 10,952,164 |  | |  | |
| Lib. 11. Non-HPLC | Leaf CTR_24 h /R3 | GGCTAC | 1,365 | 14,364,874 |  | |  | |
| Lib. 11. HPLC | Leaf CTR_24 h /R4 | ATGAGC | 1,161 | 12,210,772 |  | |  | |
| Lib. 12. Non-HPLC | Leaf CTR_24 h /R5 | CTTGTA | 1,541 | 16,181,450 |  | |  | |
| Lib. 12. HPLC | Leaf CTR_24 h /R6 | ATTCCT | 1,153 | 12,096,176 |  | |  | |
| Lib. 13. Non-HPLC | Leaf STR_24 h /R1 | AGTCAA | 1,366 | 14,368,884 |  | |  | |
| Lib. 13. HPLC | Leaf STR_24 h /R2 | CAAAAG | 1,062 | 11,152,362 |  | |  | |
| Lib. 14. Non-HPLC | Leaf STR_24 h /R3 | AGTTCC | 1,560 | 16,404,210 |  | |  | |
| Lib. 14. HPLC | Leaf STR_24 h /R4 | CAACTA | 1,338 | 14,029,986 |  | |  | |
| Lib. 15. Non-HPLC | Leaf STR_24 h /R5 | ATGTCA | 1,570 | 16,508,928 |  | |  | |
| Lib. 15. HPLC | Leaf STR_24 h /R6 | CACCGG | 1,136 | 11,933,690 |  | |  | |

CTR=Control, STR= 150 mM NaCl, _00=0 h after NaCl; _06=6 h after NaCl; _24= 24 h after NaCl.
