# Supplementary material for: Transcriptomic Analysis of Petunia hybrida in Response to Salt Stress Using High Throughput RNA Sequencing
Source: PLoS One. 2014 Apr 10;9(4):e94651. doi: 10.1371/journal.pone.0094651 (PMC3983219; doi:10.1371/journal.pone.0094651)
Supplement: Table S2 — A. Variance partitioning in Permutational Multiple Analysis of Variance (ADONIS) comparing expression profiles of the 10 most expressed transcripts between HPLC (HP) vs. non-HPLC (NH) datasets. B. Variance partitioning in Permutational Multiple Analysis of Variance (ADONIS) comparing expression profiles of the 100 most expressed transcripts between HPLC (HP) vs. non-HPLC (NH) datasets. C. Variance partitioning in Permutational Multiple Analysis of Variance (ADONIS) comparing expression profiles of the 1,000 most expressed transcripts between HPLC (HP) vs. non-HPLC (NH) datasets. D. Variance partitioning in Permutational Multiple Analysis of Variance (ADONIS) comparing expression profiles of the 10,000 most expressed transcripts between HPLC (HP) vs. non-HPLC (NH) datasets. E. Variance partitioning in Permutational Multiple Analysis of Variance (ADONIS) comparing expression profiles of the 100,000 most expressed transcripts between HPLC (HP) vs. non-HPLC (NH) datasets. (DOCX) [file pone.0094651.s004.docx]

**Supporting Information**

|  | | | | | | |
| --- | --- | --- | --- | --- | --- | --- |
| Table S2A. |  |  |  |  |  |  |
|  | Df | SumsOfSqs | MeanSqs | F.Model | R2 | Pr(>F) |
| Trt | 1 | 0.03292 | 0.03292 | 3.307 | 0.123 | 0.070 * |
| TimePoint | 2 | 0.27739 | 0.1387 | 13.935 | 0.29251 | 0.001 *** |
| Primer | 1 | 0.01079 | 0.01079 | 1.084 | 0.01138 | 0.303 |
| Trt:TimePoint | 1 | 0.42604 | 0.42604 | 42.803 | 0.44926 | 0.001 *** |
| Trt:Primer | 1 | 0.00194 | 0.00194 | 0.195 | 0.00204 | 0.805 |
| TimePoint:Primer | 2 | 0.0089 | 0.00445 | 0.447 | 0.00938 | 0.704 |
| Trt:TimePoint:Primer | 1 | 0.00122 | 0.00122 | 0.123 | 0.00129 | 0.904 |
| Residuals | 19 | 0.18912 | 0.00995 |  | 0.19942 |  |
| Total | 28 | 0.94831 |  |  | 1.000 |  |
|  |  |  |  |  |  |  |
|  |  |  |  |  |  |  |
|  |  |  |  |  |  |  |
| Table S2B. | | | | | | |
|  |  |  |  |  |  |  |
|  | Df | SumsOfSqs | MeanSqs | F.Model | R2 | Pr(>F) |
| Trt | 1 | 0.08227 | 0.08227 | 9.394 | 0.08072 | 0.004 ** |
| TimePoint | 2 | 0.36474 | 0.18237 | 20.824 | 0.35787 | 0.001 *** |
| Primer | 1 | 0.0235 | 0.0235 | 2.683 | 0.02306 | 0.066 * |
| Trt:TimePoint | 1 | 0.37505 | 0.37505 | 42.825 | 0.36799 | 0.001 *** |
| Trt:Primer | 1 | 0.00054 | 0.00054 | 0.061 | 0.00053 | 0.992 |
| TimePoint:Primer | 2 | 0.00502 | 0.00251 | 0.287 | 0.00493 | 0.936 |
| Trt:TimePoint:Primer | 1 | 0.00168 | 0.00168 | 0.192 | 0.00165 | 0.917 |
| Residuals | 19 | 0.1664 | 0.00876 |  | 0.16326 |  |
| Total | 28 | 1.01919 |  |  | 1.000 |  |
|  |  |  |  |  |  |  |
|  |  |  |  |  |  |  |
|  |  |  |  |  |  |  |
|  | | | | | | |
| Table S2C. |  |  |  |  |  |  |
|  | Df | SumsOfSqs | MeanSqs | F.Model | R2 | Pr(>F) |
| Trt | 1 | 0.12885 | 0.128852 | 15.988 | 0.13194 | 0.001 *** |
| TimePoint | 2 | 0.3752 | 0.1876 | 23.278 | 0.38421 | 0.001 *** |
| Primer | 1 | 0.02359 | 0.02359 | 2.927 | 0.02416 | 0.049 * |
| Trt:TimePoint | 1 | 0.29033 | 0.290329 | 36.025 | 0.2973 | 0.001 *** |
| Trt:Primer | 1 | -0.00009 | -0.000089 | -0.011 | -0.00009 | 1.000 |
| TimePoint:Primer | 2 | 0.0041 | 0.002052 | 0.255 | 0.0042 | 0.979 |
| Trt:TimePoint:Primer | 1 | 0.00145 | 0.001447 | 0.18 | 0.00148 | 0.952 |
| Residuals | 19 | 0.15312 | 0.008059 |  | 0.1568 |  |
| Total | 28 | 0.97656 |  |  | 1.000 |  |
|  |  |  |  |  |  |  |
|  |  |  |  |  |  |  |
|  |  |  |  |  |  |  |
|  |  |  |  |  |  |  |
|  | | | | | | |
| Table S2D. |  |  |  |  |  |  |
|  | Df | SumsOfSqs | MeanSqs | F.Model | R2 | Pr(>F) |
| Trt | 1 | 0.12885 | 0.128852 | 15.988 | 0.13194 | 0.001 *** |
| TimePoint | 2 | 0.3752 | 0.1876 | 23.278 | 0.38421 | 0.001 *** |
| Primer | 1 | 0.02359 | 0.02359 | 2.927 | 0.02416 | 0.055 * |
| Trt:TimePoint | 1 | 0.29033 | 0.290329 | 36.025 | 0.2973 | 0.001 *** |
| Trt:Primer | 1 | -0.00009 | -0.000089 | -0.011 | -0.00009 | 1.000 |
| TimePoint:Primer | 2 | 0.0041 | 0.002052 | 0.255 | 0.0042 | 0.979 |
| Trt:TimePoint:Primer | 1 | 0.00145 | 0.001447 | 0.18 | 0.00148 | 0.952 |
| Residuals | 19 | 0.15312 | 0.008059 |  | 0.1568 |  |
| Total | 28 | 0.97656 |  |  | 1.000 |  |
|  |  |  |  |  |  |  |
|  |  |  |  |  |  |  |
|  |  |  |  |  |  |  |
|  | | | | | | |
| Table S2E. |  |  |  |  |  |  |
|  | Df | SumsOfSqs | MeanSqs | F.Model | R2 | Pr(>F) |
| Trt | 1 | 0.14491 | 0.14491 | 17.6611 | 0.15436 | 0.001 *** |
| TimePoint | 2 | 0.36583 | 0.182916 | 22.2931 | 0.3897 | 0.001 *** |
| Primer | 1 | 0.02314 | 0.023138 | 2.82 | 0.02465 | 0.038 * |
| Trt:TimePoint | 1 | 0.2418 | 0.241804 | 29.4701 | 0.25758 | 0.001 *** |
| Trt:Primer | 1 | 0.00048 | 0.000479 | 0.0584 | 0.00051 | 0.997 |
| TimePoint:Primer | 2 | 0.00485 | 0.002425 | 0.2956 | 0.00517 | 0.977 |
| Trt:TimePoint:Primer | 1 | 0.00185 | 0.001845 | 0.2249 | 0.00197 | 0.943 |
| Residuals | 19 | 0.1559 | 0.008205 |  | 0.16607 |  |
| Total | 28 |  |  |  | 1.000 |  |
